# Immune-Mediated Diseases from the Point of View of Psychoneuroimmunoendocrinology

**DOI:** 10.3390/biology11070973

**Published:** 2022-06-28

**Authors:** Miguel A. Ortega, Cielo García-Montero, Oscar Fraile-Martinez, Miguel Angel Alvarez-Mon, Ana Maria Gómez-Lahoz, Guillermo Lahera, Jorge Monserrat, Roberto Rodriguez-Jimenez, Javier Quintero, Melchor Álvarez-Mon

**Affiliations:** 1 Department of Medicine and Medical Specialities, University of Alcala, 28801 Alcalá de Henares, Spain; cielo.gmontero@gmail.com (C.G.-M.); oscarfra.7@hotmail.com (O.F.-M.); maalvarezdemon@icloud.com (M.A.A.-M.); alahoz1199@gmail.com (A.M.G.-L.); guillermo.lahera@gmail.com (G.L.); jorge.monserrat@uah.es (J.M.); mademons@gmail.com (M.Á.-M.); 2 Ramón y Cajal Institute of Sanitary Research (IRYCIS), 28034 Madrid, Spain; 3 Department of Psychiatry and Mental Health, Hospital Universitario Infanta Leonor, 28031 Madrid, Spain; fjquinterog@yahoo.es; 4 Psychiatry Service, Center for Biomedical Research in the Mental Health Network, University Hospital Príncipe de Asturias (CIBERSAM), 28806 Alcalá de Henares, Spain; 5 Department of Legal Medicine and Psychiatry, Complutense University, 28040 Madrid, Spain; roberto.rodriguez.jimenez@gmail.com; 6 Institute for Health Research 12 de Octubre Hospital, (Imas 12)/CIBERSAM (Biomedical Research Networking Centre in Mental Health), 28041 Madrid, Spain; 7 Immune System Diseases-Rheumatology, Oncology Service an Internal Medicine, University Hospital Príncipe de Asturias, (CIBEREHD), 28806 Alcalá de Henares, Spain

**Keywords:** immune-mediated inflammatory diseases (IMID), psychoneuroimmunoendocrinology (PNIE), psychosomatic therapy, integrative medicine

## Abstract

**Simple Summary:**

Immune-mediated inflammatory diseases (IMIDs) are growing in prevalence and relevance in our society, entailing notable consequences for the individual and healthcare systems. These medical conditions are associated with a systemic inflammatory milieu and an aberrant functioning of the immune system, establishing a bidirectional interplay with other organs and systems of the body. Psychoneuroimmunoendocrinology (PNIE) is an area of great interest relating the immune system with the individual’s psyche, nervous, and endocrine system. As compelling evidence supports the pivotal role of PNIE in the understanding and clinical management of IMIDs, the aim of the present review is to deepen the current basic and clinical knowledge in this field.

**Abstract:**

Immune-mediated inflammatory diseases (IMIDs) represent a large group of diseases (Crohn’s, ulcerative colitis, psoriasis, lupus, and rheumatoid arthritis) evidenced by systemic inflammation and multiorgan involvement. IMIDs result in a reduced quality of life and an economic burden for individuals, health care systems, and countries. In this brief descriptive review, we will focus on some of the common biological pathways of these diseases from the point of view of psychoneuroimmunoendocrinology (PNIE). PNIE consists of four medical disciplines (psychology, nervous system, immune system, and endocrine system), which are key drivers behind the health–disease concept that a human being functions as a unit. We examine these drivers and emphasize the need for integrative treatments that addresses the disease from a psychosomatic point of view.

## 1. Introduction

### 1.1. What Are Immune-Mediated Diseases?

Immune-mediated inflammatory diseases (IMIDs) are a group of diseases with systemic inflammation and multiorgan involvement: inflammatory bowel disease (IBD) (including the subtypes Crohn’s and ulcerative colitis), uveitis, rheumatoid arthritis (RA), psoriatic arthritis (PsA), psoriasis, systemic lupus erythematosus (SLE), ankylosing spondylitis, hidrosadenitis suppurativa, sarcoidosis, atopic dermatitis (AD), connective tissue disorders, asthma, and some neurological diseases, such as multiple sclerosis (MS) [1].

In all of these multifactorial diseases, the immune system is remarkably dysregulated, and there are a number of common genetic and environmental factors despite having different clinical manifestations [2]. Pain, chronic fatigue, skin manifestations, and multiorgan dysfunction are some of the many symptoms of these diseases that reduce patients’ quality of life [3].

Epidemiological studies focus mainly on prevalence in developed countries (5–7% of people in Western society [4]), in which a lifestyle is associated with a significantly increased risk of suffering from IMIDs (e.g., poor diet, smoking habits, and emotional stress). The estimated incidence until 2019 was 80 cases per 10^5^ people per year [5]. In addition, due to the chronic disabling conditions that IMIDs impose, there is a significant socioeconomic impact and loss of productivity for individuals, health care systems, and countries. It has been estimated that the inability to perform a job properly or early retirements due to IMID are directly related to the costs involved in health care systems [6]. In 2016, for example, the estimated lifetime health costs for patients with Crohn’s and ulcerative colitis in the United States were USD 498 billion and USD 377 billion, respectively [7]. In 2006, the burden of RA in United States was EUR 42 billion, whereas in Europe it was EUR 45 billion, a mean of EUR 13,000 per patient, although the differences are notable between western Europe (EUR 17,000) and eastern Europe (EUR 5000) [8]. In Spain, point prevalence studies in 2019 highlighted a 6.4% overall prevalence of IMIDs, with psoriasis being the most frequent disease, accounting for 2.7% [9]. It is expected that the incidence worldwide will continue to increase.

### 1.2. What Is Psychoneuroimmunoendocrinology (PNIE)?

Psychoneuroimmunoendocrinology (PNIE) consists of the study of the interaction between psychic processes and the nervous, immune, and endocrine systems. That is, it is a psychobiological concept that defines the body–mind relationship in the contexts of both health and disease [10].

Pioneers, such as the psychiatrist George Salomon and the psychologist Robert Ader, first saw the relationship between the brain and the immune system and pointed out the influence of psychosocial factors on immune function. Salomon realized that patients with RA had common emotional states related to the course of the disease, and he worked with other professionals (e.g., psychologist Rudolf Moos and immunologist Alfred Amkraut) to study the effect of stressful experiences on immune function in animal models. Later, he defined PNI as *an interdisciplinary scientific field that is dedicated to the study and investigation of the mechanisms of interaction and communication between the brain (mind/behavior) and the systems responsible for the homeostatic maintenance of the organism, the nervous systems (central and autonomic), immunological and neuroendocrine, as well as their clinical implications* [11].

The concept was extended to include endocrine function. This set of four systems regulates a great variety of physiological processes whose interaction reduces the vulnerability to certain diseases. The interaction between the elements of the PNIE is explained by changes either in endocrine and neural function, which affect the immune response, or by stimulation of the immune system, which modifies endocrine and nervous function; in either case, the individual’s behaviour affects the other three systems [12]. The relevance of the body–mind balance is reflected in the high prevalence of mood disorders such as depression and anxiety associated with autoimmune, inflammatory, and metabolic diseases [13,14,15]. The inclusion of the mental factor, as an indispensable part of the process, reflects the definition of health proposed by the World Health Organization: the state of complete physical, mental, and social well-being.

### 1.3. Why Is it Important to Address the Problem of IMIDs from the Perspective of PNIE?

Immune-mediated diseases can also be understood from a psychosomatic point of view. In them we find other common denominators, such as the coexistence of psychiatric disorders in addition to metabolic ones [16]. IMIDs from the holistic point of view of the PNIE are, therefore, examples of chronic diseases that must be addressed in a multidisciplinary way by specialists in various fields of medicine. A therapy will be the more effective if the psychology of the patient is addressed to mitigate the risk of neuropathies and, likewise, the risk of associated metabolic comorbidities due to hormonal imbalance caused by the immune imbalance. That is, PNIE aims to improve the quality of life of these patients and reduce their hospital stay and reliance on medical assistance, while addressing other problems that are not as visible and preventing associated complications that worsen patients’ prognoses. Due to the aetiological similarities of genetic and environmental factors in the majority of IMIDs, it is extrapolated that there is also a common PNIE that this review intends to find.

## 2. IMIDs from the Point of View of the PNIE

### 2.1. Aetiology of IMIDs

Several studies of the immune response have determined that there are common signalling pathways in different IMIDs. Extensive genetic overlap has been found between apparently different diseases [17]. Through genome-wide association studies (GWAS), susceptibility genes and polymorphisms common to all, although also specific to each disease, have been found. Among the risk factors that can contribute to the onset of the disease, epidemiological studies have highlighted as common in all IMIDs: tobacco, poor diet, drugs, social and geographical status, microbial dysbiosis, and emotional stress [2]. That is, epigenetic factors and stressors trigger disease and alter the physiological processes of PNIE [12]. Studies in monozygotic twins have revealed environmental associations, although in cases such as type 1 diabetes (T1D), the disease is affected mainly by genetic factors in contrast to RA or scleroderma, which have a very weak association with genetic factors [18]. Due to a state of chronic inflammation, patients with IMIDs have a higher risk of cardiovascular diseases, metabolic syndrome, type 2 diabetes (T2D), and fatty liver and kidney disease [19,20]. We cannot treat the pathophysiology equally in all IMIDs. Some have a greater involvement of the intestine (IBD), the skin (psoriasis, AD), or the bones and cartilage in the joints (RA). Common biological pathways are explained, which can lead to different clinical manifestations and different organic and histological effects. The collection of information for this work has taken as a reference representative examples of IMIDs (RA, Crohn’s, ulcerative colitis, psoriasis, AD, and MS) for which there was updated scientific evidence on the index.

### 2.2. Unravelling the Key Elements of PNIE to Better Understand IMIDs

Next, part of the scientific evidence about the deregulation of the axis box that makes up the PNIE in the context of IMIDs is presented.

(a) Immune system dysfunction in IMIDs

First, as the name suggests, IMIDs are a serious dysfunction of the immune system. There is a very high expression of certain proinflammatory cytokines, such as tumour necrosis factor α (TNFα), which is why the use of cytokine blocking drugs is used to treat many of these IMIDs (IBD, uveitis, psoriasis, PAs, and juvenile idiopathic arthritis/JIA) [21]. IBD, as a representative example of IMIDs, shows a high expression of other cytokines such as interleukin 23 (IL-23) and IL-17, which are pivotal mediators of T helper 17 (Th17) responses [22]. Interestingly, the genes involved in the signalling of ILs and some related to autophagy and the innate immunity associated with IBD overlap with other diseases such as T1D [23]. In the case of psoriasis, the expression of IL-17 is enhanced, promoting proinflammatory effects, recruiting neutrophils, and prompting the expression of other proinflammatory cytokines, such as IL-6 and IL-8. In addition, they can direct the function of cytotoxic T lymphocytes (CTLs) to produce more IL-17, interferon γ (IFNγ), TNFα, and IL-21, which escape the immune suppression of regulatory T cells (Treg) [24]. The proinflammatory cytokine IL-1β also promotes Th17 polarization, as observed in psoriasis and RA, and it is suggested that it acts synergistically with IL-23 to induce the production of IL-17 by other Th and CTLs [24,25]. In addition, to highlight the exacerbated Th17 response, we can also point out the abnormal activation of Th2, Th22, and different degrees of Th1 that vary according to the AD subtype. The most characteristic cytokines in this pathology are usually IFN-γ, IL-4, IL-13, IL-31, IL-33, IL-23, IL-22, and IL-17 [26].

However, it has also been seen in RA that patients are competent in the suppression by Treg of the proliferation of other T cells, but this suppression is not effective for the exacerbated production of cytokines. To explain this mechanism, the decreased expression of Cytotoxic T-Lymphocyte Antigen 4 (CTLA-4) on Tregs, associated with a delayed immunological synapse, has been demonstrated as a key element; that is, defects in CTLA-4 lead to the deregulation of Treg function [27]. In IMIDs, an imbalance between cosignalling molecules, both costimulators (e.g., Cluster of differentiation 28 (CD28), CD40, Lymphocyte function-associated antigen 1 (LFA-1), LFA-3) and coinhibitors (CTLA-4 and programmed cell death 1 (PD-1)) has been postulated as a critical mechanism involved in psoriasis pathogenesis [28]. For these control points of immune differentiation, in several animal models of disease (e.g., RA, IBD, MS, and SLE), gene modulation has been found to regulate the expression of interleukins, TNF, CTLA-4, and IFNγ and mitigate the infiltration of lymphocytes into the affected organs [29]. The mix of cytokines (IL-1β, TNFα, and IL-6) and chemokines (IL-8), which regulate the recruitment of immune cells and inflammatory signalling mechanisms, is therefore a common network in autoimmune, inflammatory, and other IMIDs [30].

(b) Nervous system dysfunction in IMIDs

Affecting the nervous system can lead to various neuropsychiatric conditions, including mood and psychotic disorders, particularly in some autoimmune diseases with a greater involvement of nervous tissue, such as MS or SLE. Cross-sectional studies find a higher prevalence of disorders such as schizophrenia and psychosis in patients with these pathologies than in the general population due to the common genetic influence, microbiome dysfunction, autoantibodies, and immune dysregulation [31]. The chronic inflammation these patients present negatively affects the entire body, including the central nervous system (CNS) and the peripheral nervous system (PNS), projecting inflammation to these systems (neuroinflammation) [32].

In the CNS, this dysregulation of the immune system can lead to a series of alterations in the blood–brain barrier (BBB), resulting in disruptive and nondisruptive changes. In the former, the integrity of the structure is affected, while in the latter, there are changes at the level of transporters, cytokine receptors, and other inflammatory mediators. These changes will also favour transcellular migration, allowing the passage of immune cells, cytokines, and proinflammatory agents from other regions of the body, such as lipopolysaccharides (LPS) or even pathogens, resulting in the aforementioned neuroinflammation [33]. There is a third mechanism by which systemic inflammation can affect the function of the CNS known as the afferent neural pathway. Different nerves can capture and transmit inflammation, which has been described especially in the vagus nerve, although it also seems to occur in other nerves, such as the glossopharyngeal and cutaneous nerves [34]. This neuroinflammation involves important changes in the structure and long-term function of nervous system (NS) cells (neurons and glial cells). For example, there are important alterations in neuronal synapses, reducing the plasticity and development of new cells (neurogenesis), accompanied by the activation of microglia [35]. Thus, despite not being a direct cause of disease, an exacerbated neuroinflammatory response contributes significantly to the development of CNS pathologies such as Alzheimer’s disease, Parkinson’s disease, and MS [36]), resulting in a decrease in cognitive abilities [37]. In the case of the PNS, systemic inflammation also seems to affect the peripheral nerves in a very negative way, damaging their structure and leading to the development of multiple immune-mediated neuropathies [38].

The relationship between IMIDs and NS dysfunction has been established in numerous studies. On the one hand, it has been observed that patients with RA present a wide variety of nervous symptoms of different severities, from mild paresthesia to sudden death due to the impingement of the medulla oblongata by different inflammatory and compressive mechanisms that accompany the disease [39]. Patients with IBD may present peripheral and cranial neuropathies, inflammatory myopathies or myelopathies, among others, frequently leading to the appearance of MS [40]. As a whole, the evidence indicates how the physiopathology of IMIDs contributes to the accumulation of damage and alterations to the NS, with inflammation and immune dysfunction being key triggers of these events.

(c) Endocrine deregulation in IMIDs

In recent decades, attempts have been made to answer questions of great relevance regarding the relationship between the immune and endocrine systems. During the evolutionary course, a series of adaptive mechanisms were developed that linked the endocrine function with the activation of the immune system in acute inflammation processes, allocating the vast majority of energy and hormonal resources to the immune system to the detriment of other regions of the body, such as the brain or muscles [41]. Although this mechanism was an evolutionary advantage, in chronic systemic inflammation, it puts us at a disadvantage. Patients show significant endocrine alterations characterized by a decrease in and resistance to different anabolic hormones and a greater influence of catabolic hormones. For this reason, it is common for RA patients with significant systemic inflammation to develop diabetes mellitus or have insulin or IGF-1 resistance, which in turn can be regulated by inhibiting some proinflammatory cytokines, such as TNF-α [42]. Other anabolic hormones that are altered during systemic inflammation are sex hormones. In the case of androgens, it has been observed that patients with RA have lower levels of gonadal and adrenal androgens, as well as a low androgen/oestrogen ratio in both men and women, suggesting the possible role of androgens in the pathophysiology of the disease [43]. These reduced levels of testosterone can also affect the NS. For example, it has been shown that patients with Alzheimer’s disease have lower levels of free (active) testosterone and higher levels of luteinizing hormone (LH) in the blood, which in turn is associated with greater systemic inflammation compared to healthy subjects [44]. Testosterone has been shown to present a critical role in the amelioration of the exacerbated inflammatory response. Among some mechanisms proposed, testosterone seems to be important in the inhibition of adipose tissue formation and the expression of different adipokines such as leptin, TNF-α, IL-6, and IL-1, while increasing the production of adiponectin [45]. Furthermore, lowered serum testosterone may promote inflammatory processes independently of adipose tissue and age due to the increased production of inflammatory cytokines and acute phase protein production such as C-reactive protein (CRP), ferritin (FER), and alpha-1-acid glycoprotein (AAG) [46]. The prevalence of symptomatic androgen deficiency in men between 30 and 79 years of age is 5.6% and increases substantially with age [47]. Obesity, diabetes, anabolic steroid, or illicit drug use are also critically associated with low testosterone levels and pharmacological use of testosterone together with healthy lifestyle habits such as proper nutrition and exercise can aid to increase the levels of testosterone [48]. Thus, compelling evidence has found a remarkable association between low testosterone levels and IMIDs, and significant benefits from testosterone therapy in ameliorating or attenuating the symptoms of several chronic inflammatory conditions have been reported [49]. In contrast, oestrogens seem to have immunostimulatory effects, and in patients with SLE, oestrogen levels are higher than in healthy individuals, directly correlating with the severity of the disease [50].

The activation of the renin–angiotensin–aldosterone system (RAAS) and the sympathetic nervous system (SNS) will also have a notable influence on chronic systemic inflammatory processes. RAAS promotes insulin resistance and the release of norepinephrine by the SNS, which directs great energy resources to the cells of the IS [41]. The SNS will also promote the release of glucocorticoids such as cortisol under acute conditions. However, in the case of chronic systemic inflammation, glucocorticoids will be substantially altered. Due to inflammation, the adrenal glands produce insufficient levels of cortisol, with a deficit of the hypothalamic–pituitary–adrenal axis (HPA) and the adrenomedullary–sympathetic axis (SAM). This dysfunction of the HPA is explained in part by the inflammatory stimulation of hepatic cortisol production, which uncouples the central nervous system from the adrenal glands, and the effect of various cytokines that alter the activity of this axis [51]. Likewise, these defects may also be due to chronobiology, which directly influences the cyclic levels of glucocorticoids. In fact, it is known that both cortisol and melatonin, the two main chronobiological hormones, have important immunomodulatory effects and that the immune system has its own circadian rhythm, which is also influenced by other peripheral and central clocks. In turn, inflammation will also regulate these circadian rhythms and the pathways they regulate, affecting each other. All this can have important repercussions in the treatment of systemic inflammatory pathologies [52]. For instance, there is an important correlation between circadian rhythms and different IMIDs such as RA or asthma, opening promising therapeutic strategies in the treatment of these conditions [53]. In this sense, although melatonin is a mostly beneficial hormone, some studies suggest that elevated levels of this hormone during the first moments of the night in patients with RA activate the immune response, while the increase in cortisol in the early hours of the day decreases it [54]. In patients with RA, it has been reported that stiffness and functional disability are evident in the first hours of the morning due to the limited production of cortisol that becomes insufficient to inhibit the inflammatory activity initiated during the night [55]. Although more studies are needed to analyse IMIDs, such as RA, there is no doubt that chronobiology is a factor of notable relevance in the treatment of chronic inflammatory diseases and is especially important in long-term therapy with glucocorticoids [56]. Despite the weight of these mechanisms described in this neural–endocrine–immune modulation, it is necessary to understand the psychological factor as a key event of this deregulation [57].

(d) Psychology in IMIDs

It is difficult to say whether the primary cause of an individual’s ailment begins with emotional or physical stress. Humans are the sum of many parts, and we undoubtedly function as a unit in which attitude or mood can positively or negatively modulate our physiological responses. Thirty years ago, the PNI was responsible for establishing the idea that the mind has always played a key role in health and disease [58]. Since 2004, a meta-analysis has collected empirical data from more than 300 articles that demonstrate that chronic stressors are associated with the suppression of cellular and humoral immunity [59]. On the one hand, in autoimmune diseases already diagnosed or in patients being treated with immunotherapy, there has been a growing interest in knowing the aetiology and prognosis of immune dysfunction in triggering mental disorders, given the high prevalence of neuropsychiatric symptoms associated with inflammatory diseases [60]. On the other hand, in some cross-sectional studies, the social phobia caused by diseases such as IBD, RA, or MS has been evaluated through psychiatric interviews and found that depression and anxiety are greater among patients with these diseases than in the general population [61]. In both children and adults, a large number of autoimmune and immune-mediated diseases demonstrate a feedback mechanism [62]. The diseases induce psychological stress, but psychological stress induces and maintains autoimmunity. Regarding the biological effects of stress, there should be highlighted various mechanisms. On the one hand, emotional stress activates the HPA axis, leading to a systemic increase in adrenocorticotropin hormone (ACTH), glucocorticoids (cortisol), mineralocorticoids, and catecholamines [63]. These substances have important neuroimmunoendocrine effects, driving to an enhanced inflammation, gonadal axis disruption, and defects in neuroplasticity and neurogenesis, negatively influencing mood and aggravating psychiatric conditions [64]. In common autoimmune diseases in children (JIA, SLE, and T1D), psychological stress is identified as a risk or an aggravating factor [65]. The evidence indicates that the rates of anxiety and mood disorders are more prevalent in patients with other diagnosed diseases than in the general population and are sufficient to trigger or enhance the physiological state. Depression is more common in patients with IMIDs that involve very high levels of pain, such as RA. However, there is limited research on the prevalence of anxiety and depression associated with IMIDs, with limited comparison groups to distinguish a clinical picture of patients who have psychiatric disorders and IMIDs [66].

(e) The role of gut microbiota as part of the PNIE

Gut microbiota is mostly a bacterial ecosystem that affects the homeostasis of an individual and interacts with all body systems. It is important to highlight the gut–brain dialogue: the gut synthesizes elements of an individual’s diet and exerts their influence in the intestinal barrier and even in organs, such as the brain, via the BBB from the vagus nerve. These metabolites are short-chain fatty acids (SCFAs), bile acids, vitamins, metabolites derived from tryptophan and other amino acids, choline derivatives, and other peptides [67].

Some of these peptides are neurotransmitters, such as serotonin and gamma-aminobutyric acid, which modulate our behaviour and brain function. Norepinephrine also seems to be synthesized by some bacteria as a *quorum sensing* molecule. That is, we have hormones of microbial origin since the microbiota functions as an endocrine organ per se. Other pathogenic microorganisms (e.g., strains of *Escherichia coli*) can grow in the presence of other neurotransmitters, such as norepinephrine and dopamine, promoting greater intestinal motility [68].

In addition, many other peptides of low molecular weight and phenolic biochemistry, from macroantioxidants and the polyphenols of fruit and vegetables, have potent anti-inflammatory and antioxidant effects [69,70]. The decreased capacity of the microbiome in a situation of dysbiosis by IMIDs will lead to a decrease in these valuable metabolites. It is also noteworthy that the absence of many other microbial metabolites (SCFAs, secondary bile acids, or metabolites derived from tryptophan) also contributes to chronic pain in IMIDs [71]. 

In a comparative study with healthy and diseased individuals, 16S rRNA analyses identified some common and differential relative abundances of certain taxonomic groups in IMIDs. They have been identified as common components in the aetiology of IMIDs and as possible diagnostic biomarkers. For all IMIDs, the relative abundance of *Actinomyces*, *Eggerthella*, *Clostridium III*, *Faecalicoccus*, and *Streptococcus* were significantly increased, and those of *Gemmiger, Lachnospira*, and *Sporobacter* were decreased. There was also an increase in *Intestinibacter* in Crohn’s disease, *Bifidobacterium* in ulcerative colitis, and a reduction in *Coprococcus* in Crohn’s disease, *Dialister* in MS and *Roseburia* in RA [72]. However, not only the composition but also the diversity was affected. It is estimated that IBD patients harbour 25% fewer microbial genes than control individuals. In one study, the decreased richness and diversity in Crohn’s disease were more aggravated than those in other IMIDs. For ulcerative colitis, MS, and RA, a similar richness and diversity was observed among the three, and the increase in *Clostridium XIVa* and the decrease in *Coriobacteriaceae* were added to the list of previous bacterial genera [73].

Most autoimmune diseases and other IMIDs show gastrointestinal symptoms, microbial dysbiosis, intestinal hyperpermeability, and inflammation. If we add the emotional stress factor, which is an intestinal disruptor, the clinical picture is aggravated [74]. In any case, the function of the microbiome emphasizes the dependence of the other systems of the PNIE, since the microbiota–intestine–brain axis also involves the digestive endocrine function, the immune function of the intestinal mucosa, the enteric nervous system, the PNS, and the CNS [75].

### 2.3. Holistic Integration of the PNIE in IMIDs

The cytokine network not only regulates immune functions but also affects the entire organism at the metabolic, cardiovascular, and neuroendocrine levels. One characteristic of its complexity is that the interactions between different cytokines follow positive feedback loops unlike the endocrine system, which is based on negative feedback loops [76].

On the other hand, it is estimated that approximately 50% of patients with autoimmune diseases show depressive symptoms. The overactivation of the immune system is related to a disruption of circadian rhythms and the expression of clock genes, which would lead to a depressed mood [14]. In addition, several clinical trials of anti-cytokine therapy have observed that proinflammatory cytokines associated with skin diseases can coexist with depressive symptoms [13]. Higher ratios of inflammatory parameters have in fact been associated with a worse response to antidepressant treatment [77]. Both depression and chronic fatigue have been associated with increased inflammatory activation, affecting both the CNS and the PNS. The psychiatric link explains why many antidepressants decrease inflammation [16]. Many of these drugs interfere with the microbiota, which has served to recommend the use of multispecies probiotics [78]. In turn, inflammation in IMIDs implies a greater metabolic disorder, which increases the risk of suffering from diseases such as obesity or T2D, which in turn increase the incidence of depressive symptoms [79].

On the other hand, it should be mentioned here the multiple consequences of autoimmune endocrine diseases as pivotal examples of how the PNIE works in the IMID context. Different diseases are collected under this term, including common disorders such as Hashimoto’s thyroiditis, Graves’ disease, T1D, and more rare conditions such as autoimmune hypophysitis, Addison’s disease, premature ovarian failure (POF), and hypoparathyroidism [80]. In these conditions, due to the interplay between different genetic and environmental factors, the immune system produces a set of cytokines and autoantibodies affecting specific or systemic endocrine elements, eventually mediating the tissue destruction of beta pancreatic cells (T1D), thyroids (Hashimoto thyroiditis), adrenal cortexes (Addison´s disease), and so on [81]. During the course of autoimmune endocrine diseases, there is a complex interaction between the neuroendocrine and the immune system. For instance, hyperprolactinemia is frequently observed in patients with autoimmune endocrine diseases, which triggers the synthesis of IFNy and IL-2 by Th1 lymphocytes along with the activation of Th2 lymphocytes, driving to an enhanced autoantibody production [82]. In fact, prolactin is directly associated with augmented anti-DNA antibodies, islet cell antibodies (ICA), thyreoglobulin antibodies (TgAb), thyroperoxidase antibodies (TPOAb), transglutaminase antibodies (tTGAb) in SLE, type 1 diabetes mellitus, Hashimoto’s thyroiditis, and in celiac disease, respectively [83]. Moreover, there are some specific autoimmune endocrine diseases in which these processes may be better understood. For instance, in autoimmune hypophysitis, two main stages can be distinguished: (1) A subacute/acute stage characterized by the edema-infiltration of lymphocytes and plasma cells is frequently manifested with headaches and visual field alterations due to compression, together with a hyperprolactinemia and low levels of ACTH. Although these processes may be secondary to the autoimmune and inflammatory reaction, they can also be responsible for boosting the inflammatory response, driving to subclinical [84]. (2) Then, these changes can progress to a chronic stage, in which pituitary fibrosis and atrophy are accompanied by mono- or pluritropinic deficiencies with the secondary impairment of the respective target gland function. Eventually, panhypopituitarism appears in those patients as a result of persistent neuroimmunoendocrine alterations [84]. Regarding Addison´s disease, it is broadly accepted that this condition occurs as a result of the interplay between genetic and environmental factors with a central role of T lymphocytes [85]. These cells infiltrate in the adrenal cortex, leading to an extensive and progressive atrophy of the parenchyma, while the adrenal medulla remains intact. The main target of autoimmune responses are the steroidogenic enzymes implicated in the synthesis of glucocorticoids such as cortisol and mineralocorticoids (aldosterone) [86]. These endocrine changes lead to an increased production of ACTH and its precursor, pro-opiomelanocortin. In turn, these changes can lead to neurological and physical changes such as fatigue, nausea, dizziness, and weight loss, but can often be identified by specific signs such as salt-craving and changes in the skin pigmentation [87]. Besides, due to the alterations presented in these patients, other endocrine structures can be affected such as the thyroid glands, leading to autoimmune thyroid disease or the pancreas, influencing the development of type 1 diabetes mellitus [86]. An elevated hyperprolactinemia has also been observed to be directly associated with adrenocortical antibodies (ACA), supporting its relevance in Addison´s disease [83]. Likewise, all these changes can make patients with autoimmune endocrine diseases suffer from mental disorders, and in turn this could affect the neuroendocrine and immune system, sharing plenty of cellular and molecular mechanisms [88]. 

Similarly, bacterial translocation is a factor that favours sustained inflammation in IMIDs. This mechanism would also be one of the reasons why patients do not always respond adequately to available anti-inflammatory therapies, since the translocated bacterial products stimulate subsets of immune cells that produce proinflammatory cytokines [89]. In the case of autoimmunity (T1D, MS, RA, and autoimmune disease of the liver), the implementation of apoptotic mechanisms in intestinal epithelial cells in response to microbial stimuli is striking, also allowing the presentation of autoantigens and promoting the differentiation of Th17 cells [90], further worsening immunological tolerance.

**Figure 1:**
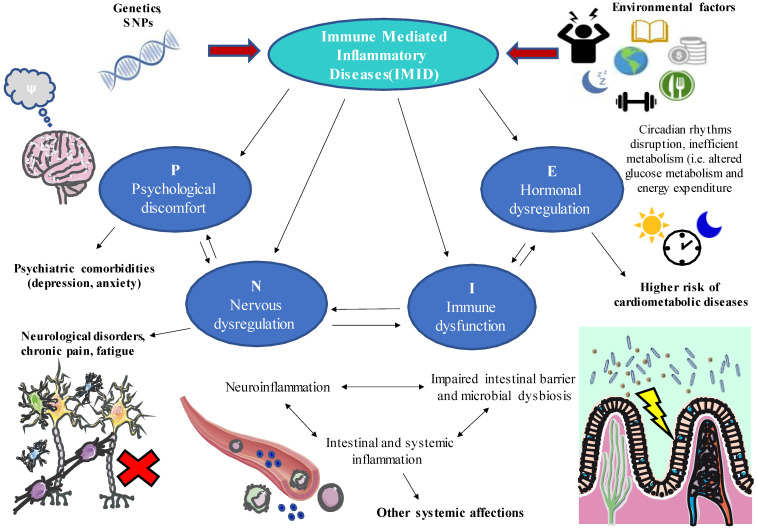
A global perspective of the role of psychoneuroimmunoendocrinology (PNIE) in immune-mediated inflammatory diseases (IMIDs). As shown, different psychological (P), neurological (N), immune (I), and endocrine (E) factors will interact significantly during IMID, which are in turn influenced by a series of environmental and genetic factors. Thus, these patients present various ailments and manifestations that go beyond the pathophysiology of the disease itself. For example, it is common to observe a systemic inflammatory response that also affects other organs, such as the brain (neuroinflammation), intestine (intestinal permeability, microbial dysbiosis), or endocrine system (alterations in glucose metabolism and energy metabolism and disruption of circadian rhythms). Other frequent consequences of IMIDs are fatigue, chronic pain, skin manifestations, psychiatric disorders, and increased risk of cardiovascular and metabolic diseases. Thus, comprehensive clinical management that considers the interrelation of all these systems is necessary to provide patient-centred treatments adapted to chronorhythms, nutrition, management of emotional stress, and physical activity.

Figure 1 shows an overview of the PNIE in IMIDs, highlighting in a synthesized way some of the ideas that we have analysed throughout the text.

## 3. IMID Treatments and Their Optimization from the PNIE

### 3.1. Available Pharmacological Treatments

Biological anti-cytokine therapy is effective in mitigating inflammation and related clinical manifestations, with optimal tolerability. The most commonly used drugs have been anti-TNFα monoclonal antibodies such as infliximab [91] and adalimumab [92], TNFα receptor antagonist inhibitors such as etanercept [93], and IL-1 receptor antagonists such as anakinra [94], which are widely used to alleviate the cytokine storm in patients with COVID-19 [95,96]. Additionally, some immunosuppressive fusion proteins, such as abatacept (CTLA4-Ig), bind to CD80 and prevent T cells from being activated [97]. Although TNFα action blockers are usually indicated, most have been applied to RA, psoriasis, PsA, Crohn’s disease, ulcerative colitis, and uveitis, to address the immunological part, with adalimumab being used for the widest range of indications [98]. The neutralization of IL-1β, as a key cytokine in the polarization of effector T cells, promoter of inflammation, and common denominator in autoinflammation and autoimmunity, has been correlated with a significant reduction in the severity of RA, T2D, uveitis, and osteoarthritis [99,100]. To control the inflammatory damage caused by several cytokines, the combination or sequential therapy of several biological agents is usually chosen, for which optimal dose models have been developed [101].

TNFα inhibitors have been shown to improve insulin sensitivity in patients with IMIDs, and it has been suggested that this may decrease the risk of T2D. During short- and long-term anti-TNFα treatment, a notable improvement in insulin resistance was observed in RA patients [102,103] and IBD patients [104]. The treatment also alleviated glycaemic control in RA patients with concurrent T2D [42].

Recently, topical and oral therapies with Janus kinase inhibitors (JAKs) have been introduced [1]; these are the initiators of the JAK/STAT pathways that mediate the transduction of inflammatory signals. They are also indicated for RA and PsA and continue to be evaluated for IBD, AD, and ankylosing spondylitis [105]. In the treatment of AD, although large-scale and long-term studies are necessary, efficacy has already been seen in the blockade of JAK, which in this case is involved in the signal transduction of multiple cytokine characteristics of the pathology (IFN-γ, IL-4, IL-13, IL-31, IL-33, IL-23, IL-22, and IL-17) [26].

In the case of RA, the known DMARDs’ (disease-modifying antirheumatic drugs) mechanism of action also interferes with the inflammatory cascade. For example, methotrexate reduces neutrophil adhesion, inhibits the local production of IL-1, and reduces the levels of IL-6 and IL-8 [106]. In addition, DMARDs, including hydroxychloroquine, methotrexate, TNFα, and IL-1β antagonists, improve glucose metabolism, while glucocorticoids in continuous use worsen glycaemic control [107]. Immunoglobulin therapy (IVIg) is also still used to neutralize autoantibodies in some IMIDs, such as lupus [108].

Although small molecules and effective biological drugs are available, they are not the only therapy to care for these patients. In addition to addressing the symptoms of chronic pain in pain units (analgesics and/or corticosteroids), it is also extremely important to address the comorbidities that accompany these diseases, since patients with IMIDs require multidisciplinary care in rheumatology and dermatology (topical and oral corticosteroids), gastroenterology, ophthalmology, and, although it is advised late or in many cases it is underdiagnosed, also in psychiatry.

### 3.2. Targeted Psychosomatic Therapy and Integrated Medicine

Immunotherapy cannot be stopped, since the restoration of immune function is impossible, and the control of inflammation must be addressed throughout the life course. Despite this, the proportion of patients who respond to the standard of care is 40–60% [109]. Increasing scientific evidence indicates that pharmacological therapy is more effective if a healthy diet accompanies it; so, it would be interesting to have the support of other professionals, such as nutritionists. Likewise, in pathologies that affect joints or cause pain, motor control exercises and active rehabilitation therapy must be considered together with physiotherapy. To contemplate the totality of the exposed aspects, psychological therapy is ultimately vital for the better management of stress, emotions, and attitudes during adversity. There is also the possibility of group therapy for anxiety or for the practice of mindfulness or mild–moderate physical activities. In the end, nonpharmacological therapy must be adapted to the needs and circumstances of the patient. The sooner action is taken at the different levels exposed, the sooner the incidence of metabolic, psychiatric, and immunological comorbidities can be prevented.

Patients with IMIDs have chronic fatigue and sleep disturbances. A higher quality of sleep, chronobiology, and stress management can contribute to well-being, in addition to a healthy diet of high scientific value, such as the Mediterranean diet [110]. In this sense, it should be highlighted the role of a great variety of foods rich in vitamins, dietary fiber, omega 3 fatty acids, and other nutraceuticals of interest, together with phenolic components of extra virgin olive oil (hydroxytyrosol, oleocanthal, and oleuropein) and aromatic spices that have been shown to have antioxidant, anti-inflammatory, and immunomodulatory properties of interest for IMIDs [111]. For instance, omega 3 polyunsaturated fatty acids (PUFAs), which are abundant in seafood and especially oily fishes have important psychoneuroimmunoendocrinological effects, favourably influencing mood and social behaviours, structural and functional properties of neurons, inflammatory response amelioration, gut microbiota, metabolic modulation, and so on [12,112]. Because of that, animal models demonstrate benefits from omega 3 PUFAs in RA, IBD, and asthma and some clinical trials of fish oil in patients with RA demonstrate remarkable benefits supported by meta-analyses of the data [113]. Despite being less studied, monounsaturated fatty acids (MUFAs), abundant in extra virgin olive oil, have also exhibited prominent anti-inflammatory effects, and its deregulation has been observed in certain neuropsychiatric conditions [114,115]. More large-scale randomized studies on these pathologies are necessary in humans to provide concrete nutritional recommendations. Currently, in addition to the Mediterranean diet, the low FODMAP (fermentable oligosaccharides, disaccharides, monosaccharides, and polyols) diet is of special interest for patients with pathologies that have greater intestinal involvement, such as IBD [116]. Additionally, as we have seen previously, due to its ability to manufacture anti-inflammatory peptides, antioxidants, and neurotransmitters, gut microbiota as a therapeutic target enhanced through diet and probiotic supplements in different IMIDs represent an interesting research topic.

Physical activity (PA) may also represent a potential support in the clinical management of several IMIDs. It is important to highlight that the effects of PA on the inflammatory markers can be different according to the type of exercises (i.e., endurance or resistance training), volume of training, intensity, and exercise duration [117]. A systematic review conducted in children and adults with chronic inflammatory diseases showed that whereas acute exercise training were associated with increased levels of inflammatory markers, chronic endurance exercise programs can attenuate systemic inflammation in certain conditions [118]. Likewise, another recent systematic review observed that intense long exercise can lead, in general, to higher levels of inflammatory mediators, but in contrast, moderate exercise or vigorous exercise with appropriate resting periods can achieve maximum anti-inflammatory benefits [119]. In this context, when the training program is adapted, patients with rheumatic diseases exhibit decreased levels of IL-6, CRP, and the number of T CD4 lymphocytes may decrease after moderate exercise in the synovial fluid of patients with RA [117]. Exercise promotes the production of myokines, an endocrine product released my muscle cells with potential anti-inflammatory properties, explaining its benefits in patients with IMIDs [120]. Interestingly, myokines can also positively influence the adipocyte function, the gut microbiota, and the endocrine system [121,122], as well as the brain and neuropsychiatric function [123]. Thus, myokines and exercise can positively influence the entire components of the PNIE, eliciting the central actions of exercise in patients with IMID. Further studies are however required to describe adequate training programs for patients with inflammatory diseases, being an object of study in current clinical trials. 

It is worth mentioning, both for IMIDs and other multifactorial diseases, it is important that psychosomatic medicine be accompanied by psychotherapy. Thus, the emotional and mental impact of physical symptoms are analysed, and the full awareness and willpower of the individual are emphasized as key to a patient’s well-being. Psychosocial interventions can also favourably modulate the immune system function and reduce inflammatory markers [124,125], showing the potential role of these approaches in the management of IMIDs. Finally, sleep-based interventions, especially cognitive–behavioral therapy for insomnia (CBTi) has been widely explored in patients with IMIDs with concomitant sleep disturbances [126]. This strategy includes relaxation, sleep restriction, stimulus control, and cognitive techniques [127]. Other approaches are melatonin or hypnotic drugs such as benzodiazepines or antidepressants for patients with depression, although they are less effective and may show some adverse effects for these patients [126]. It would be interesting to conduct studies in this field in an effort to improve the prognosis and quality of life of patients with IMIDs. 

## 4. Conclusions

IMIDs are a large group of chronic inflammatory diseases that impair quality of life and must be understood from the psychosomatic point of view. In this brief review, we have reviewed the importance of the interaction of various branches of medicine that converge in these pathologies, the PNIE, understanding that the approach of these patients deserves to be multidisciplinary since the body functions as a unit and different systems interact with others.

**Figure 2:**
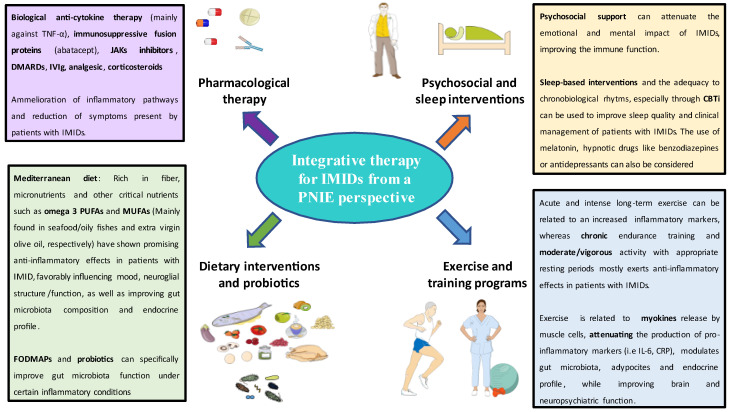
A holistic view of the potential treatment of IMIDs from a PNIE perspective.

We also note that IMIDs are a great example of so many multifactorial diseases for which integrative medicine and intervention in lifestyle factors or in certain cases with genetic risk factors can be acted on in a timely manner, prior to the triggering of advanced stages of immune dysfunction. There is increasing scientific evidence that demonstrates and reinforces that the key to our health is found in the balance of these factors (Figure 2). In this way, a decrease in treatment time and health care costs would be possible.

A holistic view of the PNIE is that all physical illness entails mental illness. Together with the concept of psychosomatic therapy, this reminds us of the famous physician and philosopher Hippocrates (460–370 BC), who said, “The natural force within each of us is the greatest healer of all”.

## Data Availability

Not applicable.
